# Transcranial Direct Current Stimulation of the Right Lateral Prefrontal Cortex Changes *a priori* Normative Beliefs in Voluntary Cooperation

**DOI:** 10.3389/fnins.2018.00606

**Published:** 2018-08-31

**Authors:** Jianbiao Li, Xiaoli Liu, Xile Yin, Shuaiqi Li, Pengcheng Wang, Xiaofei Niu, Chengkang Zhu

**Affiliations:** ^1^China Academy of Corporate Governance, Reinhard Selten Laboratory, Business School, Nankai University, Tianjin, China; ^2^Department of Economics and Management, Nankai University Binhai College, Tianjin, China; ^3^School of Business Administration, Zhejiang Gongshang University, Hangzhou, China; ^4^Business School, Tianjin University of Finance and Economics, Tianjin, China

**Keywords:** *a priori* normative beliefs, voluntary cooperation, identity, rLPFC, transcranial direct current stimulation

## Abstract

*A priori* normative beliefs, the precondition of social norm compliance that reflects culture and values, are considered unique to human social behavior. Previous studies related to the ultimatum game revealed that right lateral prefrontal cortex (rLPFC) has no stimulation effects on normative beliefs. However, no research has focused on the effects of *a priori* belief on the rLPFC in voluntary cooperation attached to the public good (PG) game. In this study, we used a linear asymmetric PG to confirm the influence of the rLPFC on *a priori* normative beliefs without threats of external punishment through transcranial direct current stimulation (tDCS). Participants engaged via computer terminals in groups of four (i.e., two high-endowment players with 35G$ and two low-endowment players with 23G$). They were anonymous and had no communication during the entire process. They were randomly assigned to receive 15 min of either anodal, cathodal, or sham stimulation and then asked to answer questions concerning *a priori* normative beliefs (norm.belief and pg.belief). Results suggested that anodal/cathodal tDCS significantly (*P* < 0.001) shifted the participants’ *a priori* normative beliefs in opposite directions compared to the shift in the sham group. In addition, different identities exhibited varying degrees of change (28.80–54.43%). These outcomes provide neural evidence of the rLPFC mechanism’s effect on the normative beliefs in voluntary cooperation based on the PG framework.

## Introduction

Neuroscience studies on social norms prove that the human brain may have potential cognitive and neural processes that underlie the ability to learn norms, follow norms, and enforce norms by generating appropriate behavioral responses to social norm compliance and normative judgments (Güth et al., 1982; Montague and Lohrenz, 2007; Buckholtz and Marois, 2012; Liu et al., 2017). For example, Buckholtz and Marois (2012) suggested a potential neurobiological architecture that may underpin norm learning, norm compliance, and norm enforcement (social sanctions or internal sanctions). They found that a dorsal frontostriatal circuitry is essential for integrating information about sanction threats into decision-making to incentivize norm-compliant behavior. Whether or not the induction of right lateral prefrontal cortex (rLPFC) can change *a priori* normative beliefs in a controlled behavioral voluntary contribution paradigm has not been investigated in the context of social norm compliance. Therefore, changing *a priori* normative beliefs under controlled experimental conditions in healthy volunteers is necessary to clarify causally the role of rLPFC in voluntary cooperative behaviors.

Human beings are the most social creatures among all species known, because none of the other species share our capacity for stable large-scale cooperation among genetically unrelated individuals. This unique feature of human culture is made possible by cognitive capacities that permit us to establish, transmit, and enforce social norms (Fehr and Fischbacher, 2004; Buckholtz and Marois, 2012; Yin et al., 2017). A social norm is a behavioral rule that is enforced by social sanctions (Coleman, 1990) and internal sanctions (e.g., feeling of guilt) (Lindbeck, 1997). “One should not litter” is an example of a social norm. Many people do not litter even when they know that nobody is observing them because people have subjective perceptions of norms, and these subjective perceptions can guide the opinions of individuals (Buckholtz and Marois, 2012). In the context of social norms, the average person does not know the actual rates of behaviors or opinions in their community (Tankard and Paluck, 2016). As they have unreliable information about what others actually think, they need to infer what (e.g., thoughts, beliefs, desires, intentions, and motivations) is going on inside other people’s heads. This subjective inference is defined as “*a priori* normative beliefs.”^1^ *A priori* normative beliefs are *a priori* beliefs based on perception of other people’s social norms and are a reference point that guides people’s behavior in social cooperation. The cooperative behaviors and actions of subjects are thought to rely strongly on the *a priori* normative beliefs in charge of regulating and coordinating thoughts and motivations under norm enforcement. Hence, extensive debates persist regarding deep neural insights into *a priori* normative beliefs and the manner of their implementation in the brain (Ruff et al., 2013; Sanfey et al., 2014).

The result of a long stream of laboratory experiments related to voluntary contributions in public good (PG) environments has already been established solidly. In the basic PGs, participants secretly decide how much of their endowment contribute into a public pool and how much remain. Contributions in the public pool, which are multiplied by a factor (greater than one and less than the number of players), are evenly divided among all participants. The actual level of contributions, which usually ranges between 40 and 60% of the total endowment (Chaudhuri et al., 2016), depends on various factors, such as the number of players and the *per capita* rate of return of the PG relative to that of the private good (Keser and Winden, 2000). Currently, although no agreement has been reached about why subjects contribute, an influential explanation is conditional cooperation. Conditional cooperation can be considered as a motivation on its own or a consequence of some fairness preferences, such as “altruism,” “warm glow,” “inequity aversion,” or “reciprocity” (Fischbacher et al., 2001). Experiments on conditional cooperation found that subjects usually contribute similarly to their co-players (Keser and Winden, 2000; Brandts and Schram, 2004; Kocher et al., 2007; Spiller et al., 2016) and are willing to contribute to a PG when others also contribute or are expected to do so (Fischbacher and Gächter, 2010). For example, the studies of Fischbacher and Gächter (2010) on conditional cooperation indicate that individual cooperation often depends on whether a person thinks others cooperate. The existence and extent of conditional cooperation are considerably influenced by the beliefs elicited on the subjective perception of norms (e.g., people contribute nothing because they believe others will contribute nothing, Kocher et al., 2008). Two possible situations are considered before a decision is made. On the one hand, some subjects must at least know of social norms and follow them (Elster, 1989; Bicchieri, 2006). On the other hand, participants may feel that their partners may not follow a norm even if it exists (Reuben and Riedl, 2009; Spiller et al., 2016). In either case, the subject needs to infer from the belief of others. Thus, the ability to attribute thoughts to others and infer their mental states plays a crucial role in social interactions (Sellaro et al., 2015).

According to the definition of Spiller et al. (2016), belief to infer “what others do” is a kind of *a priori* normative beliefs. Previous studies provided evidence by showing that people contribute more to a PG when they expect others to contribute more as well (Kachelmeier and Shehata, 1997; Croson, 2007). On the basis of these views, we may conjecture that subjects tend to follow their *a priori* normative beliefs concerning contributions. That is, subjects consider the actions of others whom they inferred as a reference for their own behavior. In this case, the essence of *a priori* normative beliefs is a reference point that is formed in the context of common knowledge considered as a “norm.” This description indicates that *a priori* normative beliefs play a key role in judging others’ motives and are the basis of a subject’s action in cooperation.

Human societies enforce norm by threatening norm violators with sanctions (social or internal) (Coleman, 1990; Lindbeck, 1997; Eriksson et al., 2017). Neuroscience studies on norms have mostly focused on the neural basis of sanctions (Sanfey et al., 2003, 2014; Spitzer et al., 2007; Boksem and De Cremer, 2010; Ruff et al., 2013; Xiang et al., 2013). All these studies used sanctioned cooperation based on the ultimatum game (UG). The UG consists of two players: proposer and responder. The proposer decides how much of a monetary endowment to split with the responder, while the responder could accept the offer or, if he/she deems the offer as violating a social norm, reject it (Ruff et al., 2013; Sanfey et al., 2014). These studies proved that the human brain has developed neural processes to support social cooperation by punishing norm violations, which are also important in sustaining human cooperation in the PG (Fehr and Fischbacher, 2004; Reif et al., 2017).

Sanfey et al. (2003) used functional magnetic resonance imaging of UG players, who responded by complying with or violating the social norm, to investigate the neural substrates of cognitive processes involved in economic decision-making. In the study, behaviors who violated the social norm elicited activity in brain areas related to the dorsolateral prefrontal cortex (DLPFC). Spitzer et al. (2007) also found that the increase in norm compliance of individuals exhibit a strong positive correlation with activations in the right DLPFC. Similarly, a lesion of the ventromedial prefrontal cortex increases the rate of rejections of offers that violate social norms in the UG (Koenigs and Tranel, 2008). Studies on non-invasive brain stimulation [e.g., transcranial direct current stimulation (tDCS)] likewise found that interfering with the activity in the DLPFC decreases the rate of rejections (Van’t et al., 2005). Mounting evidence from neuroimaging and lesion studies suggests that the DLPFC is associated with social norm violations (Aron et al., 2014; Hardung et al., 2017). Recently, the prefrontal cortex (PFC) was proven to be central to higher-level cognition (Aron et al., 2007; Azuar et al., 2014; Bahlmann et al., 2015; Nee and D’Esposito, 2016). Nee and D’Esposito [(2016), p. 17] stated that “caudal lateral prefrontal cortex (LPFC) was involved in current processing, providing selective attention to visual stimulus features, while rostral LPFC was involved in future processing, enabling the retention of information for integration into future processing. The mid LPFC appeared to synthesize both current and future processing allowing the use of current and future informed contextual information to organize behavior.” In addition, an area in rLPFC is activated during a norm-compliant behavior triggered by social punishment threats (Spitzer et al., 2007), an activation that changes the social cooperation among participants (Ruff et al., 2013; Sanfey et al., 2014; Liu et al., 2017). Therefore, rLPFC, which is necessary for norm-compliant behaviors and enable humans to anticipate sanctions for norm violations and distinguish “right” from “wrong” (Ruff et al., 2013; Liu et al., 2017), is a key biological prerequisite for an evolutionarily and socially important aspect of human behavior, and its activity exerts a particularly strong effect on social cooperation.

Decision-making in social dilemmas is suggested to rely on the relative judgment of two or more alternatives and individual factors affecting judgments and decisions. (Ramsøy et al., 2015; Liu et al., 2017). Previous research proved that the tDCS of rLPFC leads to a change in the norm judgment based on voluntary cooperation (Liu et al., 2017). The results suggested that anodal/cathodal tDCS increases/decreases participants’ judgment of “right contribution” (i.e., the amount individual ought to contribute) in opposite directions unlike in the sham group. Spiller et al. (2016) proved that *a priori* normative beliefs were also influenced by the “right contribution.” Relying on the results and analyses presented above, we can conjecture that if *a priori* normative beliefs are influenced by the norm in other people’s heads, then stimulating the same brain region (i.e., rLPFC) should also affect the *a priori* normative beliefs. Accordingly, we assume that if anodal/cathodal tDCS is applied to increase/decrease the activities of the rLPFC, the participants’ *a priori* normative beliefs will be changed. Specifically, anodal tDCS will improve the *a priori* normative beliefs, whereas cathodal tDCS will deteriorate it.

Our analysis focused on two broad categories of beliefs and brain regions that are important for *a priori* normative beliefs as revealed in previous studies (Adolphs, 2009; Fishbein and Icek, 2010; Spiller et al., 2016). To provide neural evidence of *a priori* normative beliefs among different identities, we used tDCS to investigate whether the increase or decrease of rLPFC excitability among healthy participants influences *a priori* normative beliefs in voluntary cooperation. We expected that the induction of the rLPFC by applying tDCS causes a significant change in the contribution of *a priori* normative beliefs compared with that in the sham group and that treatment effects can be observed.

## Materials and Methods

### Subjects

The subjects of this experiment were the same as Liu et al. (2017) and Li et al. (2018). A total of 83 healthy subjects (recruited from Nankai University students; 41 females and 42 males ranging from 20 to 30 years old) were kept in the sample. None of them had suffered from any neurological or psychiatric disorders. One participant in the anodal stimulation treatment felt discomfort, and we terminated the experiment. Participants randomly divided into three treatments, namely, cathodal (*n* = 28, 12 males), anodal (*n* = 27, 18 males), and sham (*n* = 28, 12 males) stimulation. All the participants had no ex-ante knowledge of neurological (tDCS) or PG tasks, and all voluntarily joined this study with informed consents. The experiment was performed in accordance with the Declaration of Helsinki and was approved by the Ethics Committee of Business of Nankai University. All these 83 participants reported no adverse side effects (e.g., pain on the scalp or headaches) after the experiment.

### Transcranial Direct Current Stimulation

The tDCS of the human motor cortex induces shifts in cortical excitability during and after stimulation under the electrode (Batsikadze et al., 2013; Jamil et al., 2017). These shifts are polarity-specific, with cathodal and anodal tDCS usually resulting in a decrease and an increase in cortical excitability, respectively (Iyer et al., 2005; Nitsche et al., 2008; Utz et al., 2010; Kadosh, 2013). Unilateral (Brückner and Kammer, 2017; Luo et al., 2017) and same effects exist (Marshall et al., 2005; Filmer et al., 2015) as well, although the latter is less common than the former. tDCS has become a kind of research paradigm in neural science. Thus far, brain stimulation studies in humans mostly show unidirectional maladaptive effects on decision-making, rendering participants more impulsive, selfish, or cognitively biased (Knoch et al., 2006; Chang and Sanfey, 2013; Ruff et al., 2013).

**FIGURE 1:**
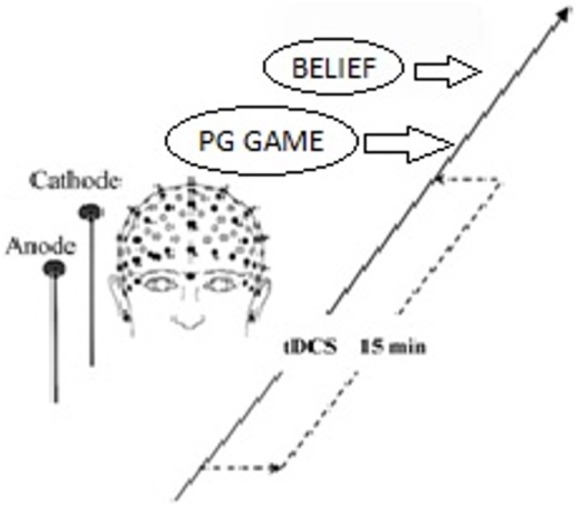
Schematic representation of the experimental design. After 15 min of stimulation, each participant decided the amount of contribution. After that, they answered questions including two pg.belief questions and two norm.belief questions.

On the basis of this finding and the general role of rLPFC in behavior control (Miller and Cohen, 2001; Aron et al., 2004), we randomly sorted participants into three stimulation groups, in which the neural excitability in the rLPFC was enhanced with anodal tDCS, reduced with cathodal tDCS, or left unaltered by sham tDCS as control for possible non-neural effects of stimulation. All participants received tDCS delivered by a battery-driven stimulator (Neuro Conn, Germany) in our experiment. tDCS was applied using a set of standard 5 cm × 7 cm electrodes fixed with rubber straps, which is the most commonly used approach in tDCS (Fusco et al., 2013; Li et al., 2017). For subjects receiving tDCS, the anodal/cathodal electrode was placed over the rLPFC according to the international EEG 10–20 electrode system, and the reference electrode (cathode for anodal tDCS and anode for cathodal tDCS) was positioned over the vertex, which was consistent with the design of Ruff et al. (2013). The stimulation current was constant at 1.0 mA intensity (Ambrus et al., 2012; Meesen et al., 2014) with 15 s of ramp up and down. Participants in the anodal/cathodal group first received 15 min of stimulation. After that, the experimental task began immediately. They were requested to complete a self-report on *a priori* normative beliefs. (Schematic representation of the experimental design, see **Figure 1**) The procedures were the same for the sham group, except that the current was stopped after the first 30 s. The 30-s stimulation in the sham condition can mimic the itching sensation of real stimulation without producing any significant neural-altering effects on the cortex (Civai et al., 2015; Willis et al., 2015; Li et al., 2017). The protocol was approved by the Ethics Committee of Business of Nankai University, and all participants gave written informed consent.

### Task and Procedure

The experimental task we conducted in the experiment was similar to those conducted by Spiller et al. (2016), except that tDCS was applied to the subjects before they participated in the experimental task. In the experiment, the participants engaged in anonymous social interactions with actual financial consequences via computer terminals. The unit of payoff in the experiment was game dollar (G$), and the exchange ratio was 1G$ = 1.5 Chinese Yuan (RMB). Payments were exchanged to cash after the experiment. The average duration was 60 min with payments of approximately 50RMB (7–8$).

Subjects played a linear PG in groups of four players, two HIGH players (A1, A2) with endowments of 35G$ and two LOW players (B1, B2) with endowments of 23G$ that were asymmetric. Endowments were chosen so that 50% contributions were not an integer and not near a multiple of 5 to reduce the attraction potential of focal points (Spiller et al., 2016).

The payoff function of PG was π_*i*_ = *X*_*i*_ - *x*_*i*_ + 0.6 $\sum_{i=1}^{4}$ *x*_*i*_, where *X*_*i*_ was the endowment, *x*_*i*_ was the contribution, and $\sum_{i=1}^{4}$ *x*_*i*_ was the sum contributions of participants from the same group. At the beginning of each trial, the subjects were informed of their identity types (A1, A2, B1, and B2). Then they were asked to answer questions related to beliefs about themselves, voluntary cooperative level and beliefs about others. We did not focus on the beliefs about themselves and voluntary cooperative level in the current study. However, we have emphatically discussed them in Liu et al. (2017) and Li et al. (2018), respectively. In this paper, we focused on the beliefs about others which were tested by pg.belief questions and norm.belief questions:

pg.belief questions: How much do you believe your peers will contribute? If they are HIGH players (A1 or A2) and Low players (B1 or B2), respectively.

norm.belief questions: How much do you believe your peers on average think is the “right” contribution? If they are HIGH players (A1 or A2) and Low players (B1 or B2), respectively.

In each trial, the identity types of subjects were reassigned and endowments were started from the initial situation. A total of 16 trials were conducted. We assigned fixed orders (pseudorandom order) in which all identities were assigned to avoid the order effect. The subjects knew neither how many trials they would play nor any feedback about contributions and payoff.

In addition to the payoff from the contribution and non-contributed endowment, subjects were also told they could receive additional incentives, which were higher if their beliefs were closer to the actual mean of group contributions in the two pg.belief questions. For example, if the bias was less than 1G$, then they would earn 4RMB.

### Statistical Analyses

The levels of beliefs were assessed using mean values (the beliefs asked during the experiment). Two types of beliefs were tested: (1) pg.belief (How much do you believe your peers will contribute?) and (2) norm.belief (How much do you believe your peers on average think is the “right” contribution?). Three treatment of tDCS stimulation groups were formed: (1) anodal, (2) sham, and (3) cathodal. The PG had two types of players, namely, (1) HIGH (35G$, A1 and A2) and (2) LOW (23G$, B1 and B2), with two types having four pairs of players: (1) HIGH for HIGH (indicates HIGH players to the question for HIGH players), (2) LOW for HIGH (indicates LOW players to the question for HIGH players), (3) HIGH for LOW (indicates HIGH players to the question for LOW players), and (4) LOW for LOW (indicates LOW players to the question for LOW players).

The levels of the two types of beliefs (norm.belief and pg.belief) were first evaluated using two-way ANOVA: 2 (types of players: HIGH and LOW) × 3 (tDCS stimulation groups: anodal, sham, and cathodal). One-way ANOVA was then performed to test the difference of norm.belief and pg.belief in three stimulation groups, respectively. Moreover, the mean levels of norm.belief and pg.belief between stimulation group and sham group were evaluated using *t*-test and rank-sum test. We also considered four pairs of players and conducted two-way ANOVA: 4 (pairs of players: HIGH for HIGH, HIGH for LOW, LOW for HIGH, LOW for LOW) × 3 (tDCS stimulation groups: anodal, sham, and cathodal).

## Results

### Behavioral Data

We analyzed the mean values of the participants with different endowments among the three stimulation groups (**Table 1**). Results showed that the participants were sensitive to their endowment. For one thing, both HIGH and LOW players believed a higher “right” average contribution (norm.belief) relative to that of the HIGH players than to that of the LOW players. Furthermore, the players with the same initial endowment had a higher expectation of their peers (pg.belief) than those with different initial endowments, except for the pg.belief relative to LOW players in the cathodal group (8.71 < 8.89).

**Table 1:** Mean values of norm.belief and pg.belief.

Stimulation groups Pairs of player types	norm.belief			pg.belief		
	Anodal	Sham	Cathodal	Anodal	Sham	Cathodal
HIGH for HIGH	30.89 (6.07)	19.89 (5.05)	13.11 (6.51)	27.63 (9.00)	18.57 (5.48)	12.07 (5.58)
LOW for HIGH	29.18 (7.80)	20.96 (7.15)	14.64 (7.50)	28.74 (8.52)	18.61 (7.00)	13.25 (5.89)
HIGH for LOW	21.42 (3.08)	15.04 (4.06)	9.75 (6.08)	19.81 (5.68)	14.14 (4.37)	8.71 (5.08)
LOW for LOW	20.26 (4.42)	13.93 (4.83)	11.04 (5.97)	17.96 (6.37)	13.25 (5.67)	8.89 (4.55)

Mean values of norm.belief and pg.belief in three stimulation groups. SDs are enclosed in parentheses. Column “Pairs of player types” indicates which player type the answer was provided (e.g., norm.belief of anodal in row “HIGH for LOW” indicates the mean response of HIGH players (in the anodal stimulation group) to the question *How much do you believe your peers on average think is the “right” contribution?* for LOW players B1 and B2?).

### General Effect of tDCS Over rLPFC on *a priori* Normative Beliefs

**FIGURE 2:**
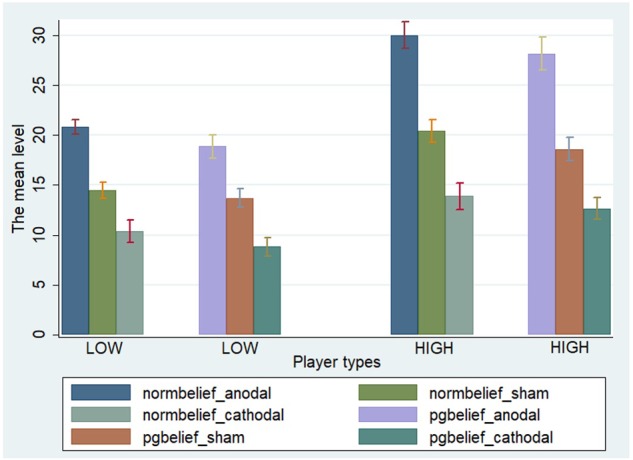
*A priori* normative beliefs in three stimulation groups. Mean values of norm.belief and pg.belief in the three stimulation groups (anodal, sham, and cathodal) between two types of players (HIGH and LOW).

We performed two-way ANOVA for norm.belief with the stimulation type (anodal, cathodal, and sham stimulation) as a between-subject factor and the player type (HIGH and LOW) as a within-subject factor. Significant main effects of stimulation type [*F*_(2,329)_ = 138.38, *P* < 0.001] and player type [*F*_(1,330)_ = 89.04, *P* < 0.001] were noted. Importantly, a significant interactive effect of stimulation type and player type was found [*F*_(2,329)_ = 6.58, *P* = 0.002]. We also performed two-way ANOVA for pg.belief with the stimulation type (anodal, cathodal, and sham stimulation) as a between-subject factor and the player type (HIGH and LOW) as a within-subject factor. Significant main effects of stimulation type [*F*_(2,329)_ = 114.51, *P* < 0.001] and player type [*F*_(1,330)_ = 74.83, *P* < 0.001] were likewise observed. A significant interactive effect of stimulation type and player type [*F*_(2,329)_ = 5.93, *P* = 0.003] was obtained (**Figure 2**).

**FIGURE 3:**
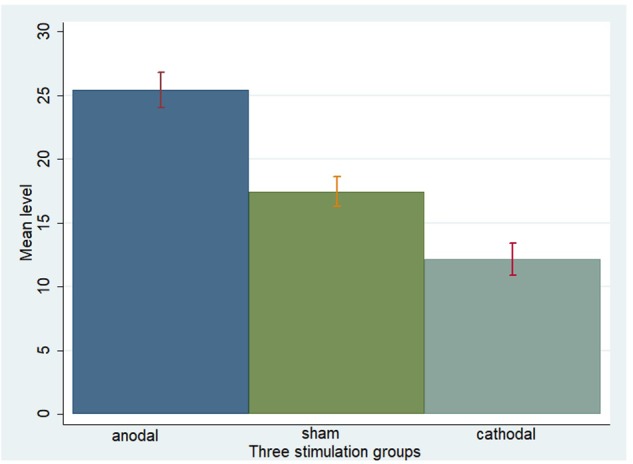
Norm.belief in three stimulation groups. Mean values of norm.belief in the three stimulation groups (anodal, sham, and cathodal) of all players.

**FIGURE 4:**
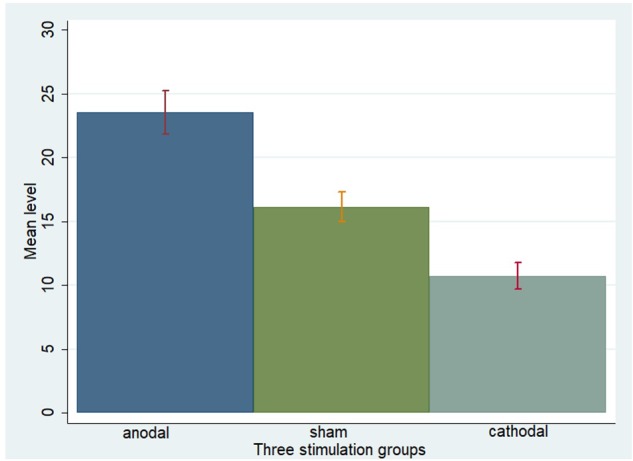
Pg.belief in three stimulation groups. Mean values of pg.belief in the three stimulation groups (anodal, sham, and cathodal) of all players.

One-way ANOVA, Kruskal–Wallis test, *t*-test, and rank-sum test were used to analyze the difference among the *a priori* normative beliefs (norm.belief and pg.belief) of the three stimulation groups. The current data show that the mean levels of norm.belief of the anodal, sham, and cathodal groups were 25.44 (SD = 7.26), 17.46 (SD = 6.13), and 12.13 (SD = 6.72), while the mean levels of pg.belief were 23.54 (SD = 8.80), 16.14 (SD = 6.15), and 10.73 (SD = 5.59), respectively. Significant differences were observed in the norm.belief and pg.belief values of the three stimulation groups [*F*_(2,329)_ = 109.17, *P* < 0.001; Kruskal–Wallis test *P* < 0.001 and *F*_(2,329)_ = 93.53, *P* < 0.001; Kruskal–Wallis test *P* < 0.001, respectively]. The mean levels of norm.belief and pg.belief in the anodal stimulation group were significantly higher than those in the sham stimulation group (*t* = 8.824, *P* < 0.001; *Z* = 8.031, *P* < 0.001 and *t* = 7.245, *P* < 0.001; *Z* = 7.073, *P* < 0.001, respectively, for the *t*-test and rank-sum test). The mean level of the cathodal stimulation group was significantly lower than that of the sham stimulation group (*t* = 6.190, *P* < 0.001; *Z* = 6.294, *P* < 0.001 and *t* = 6.888, *P* < 0.001; *Z* = 6.571, *P* < 0.001, respectively, for the *t*-test and rank-sum test; **Figures 3, 4**).

### Effect of tDCS Over rLPFC on *a priori* Normative Beliefs of Asymmetric Identity

We compared the level of norm.belief and pg.belief among the four pairs of players under three stimulation groups. We conducted two-way ANOVA: 4 (pairs of players: HIGH for HIGH, HIGH for LOW, LOW for HIGH, LOW for LOW) × 3 (tDCS stimulation groups: anodal, sham, and cathodal). Significant main effects of stimulation groups [*F*_(2,329)_ = 137.64, *P* < 0.001; *F*_(2,329)_ = 114.29, *P* < 0.001] and the pairs of players [*F*_(3,328)_ = 29.60, *P* < 0.001; *F*_(3,328)_ = 25.35, *P* < 0.001] to norm.belief and pg.belief were noted, respectively. Significant differences were observed, and the following results were found: norm.belief HIGH for HIGH [*F*_(2,80)_ = 63.36, *P* < 0.001; Kruskal–Wallis test *P* < 0.001], norm.belief HIGH for LOW [*F*_(2,80)_ = 44.28, *P* < 0.001; Kruskal–Wallis test *P* < 0.001], norm.belief LOW for HIGH [*F*_(2,80)_ = 26.06, *P* < 0.001; Kruskal–Wallis test *P* < 0.001], norm.belief LOW for LOW [*F*_(2,80)_ = 23.24, *P* < 0.001; Kruskal–Wallis test *P* < 0.001], pg.belief HIGH for HIGH [*F*_(2,80)_ = 35.66, *P* < 0.001; Kruskal–Wallis test *P* < 0.001], pg.belief HIGH for LOW [*F*_(2,80)_ = 33.03, *P* < 0.001; Kruskal–Wallis test *P* < 0.001], pg.belief LOW for HIGH [*F*_(2,80)_ = 32.70, *P* < 0.001; Kruskal–Wallis test *P* < 0.001], and pg.belief LOW for LOW [*F*_(2,80)_ = 18.22, *P* < 0.001; Kruskal–Wallis test *P* < 0.001].

**FIGURE 5:**
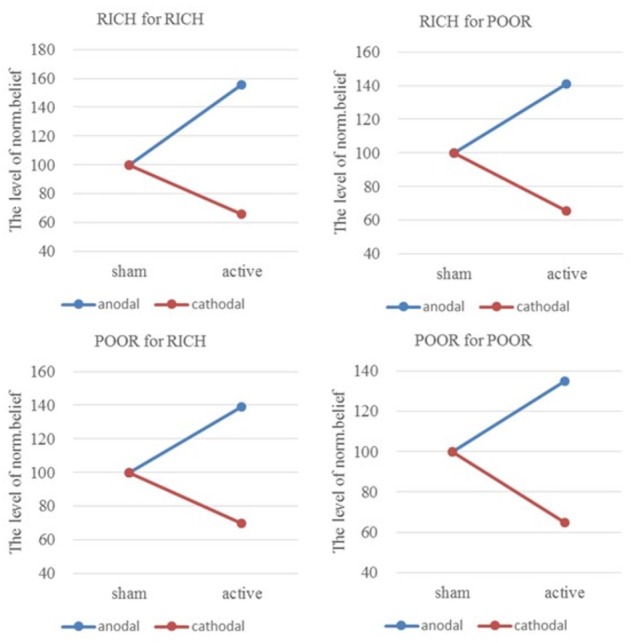
Ratios of norm.belief of four pairs of players. From sham to stimulation, the ratios of individual norm.belief change increased by 55.30% (HIGH to HIGH), 41.13% (HIGH to LOW), 39.27% (LOW to HIGH), and 34.71% (LOW to LOW) in the anodal group, and the matching ratios were attenuated by 34.09, 34.41, 30.15, and 35.17% in the cathodal group, respectively.

From sham to stimulation, the ratios of individual norm.belief change increased by 55.30% (HIGH to HIGH), 41.13% (HIGH to LOW), 39.27% (LOW to HIGH), and 34.71% (LOW to LOW) in the anodal group, and the matching ratios were attenuated by 34.09, 34.41, 30.15, and 35.17% in the cathodal group, respectively (**Figure 5**). The difference in improvement percentage of norm.belief among three stimulation groups with the same identities (HIGH for HIGH and LOW for LOW) is significant [*F*_(2,163)_ = 74.03, *P* < 0.001; Kruskal–Wallis test *P* < 0.001]. The difference among groups with different identities (HIGH for LOW and LOW for HIGH) is also significant [*F*_(2,163)_ = 25.26, *P* < 0.001; Kruskal–Wallis test *P* < 0.001]. This result means that the same stimulus has different effects on people of different identities.

**FIGURE 6:**
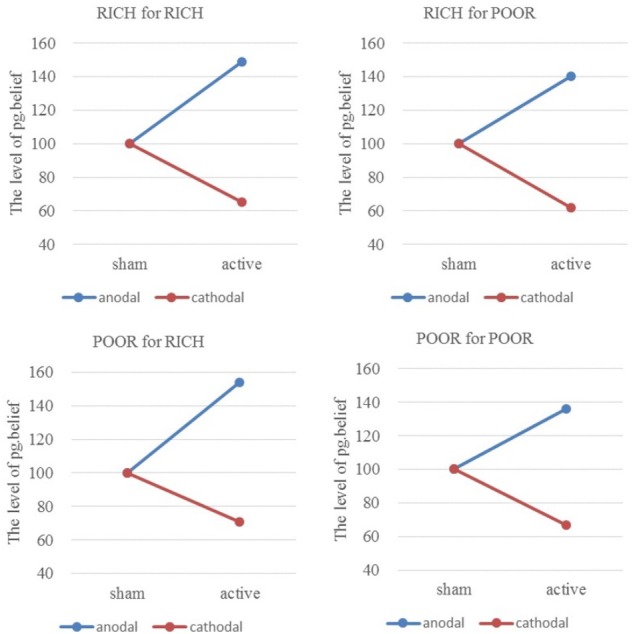
Ratios of pg.belief of four pairs of players. From sham to stimulation, the ratios of individual pg.belief change increased by 48.79% (HIGH to HIGH), 40.10% (HIGH to LOW), 54.43% (LOW to HIGH), and 35.55% (LOW to LOW) in the anodal group, and the matching ratios were attenuated by 35.00, 38.40, 28.80, and 32.91% in the cathodal group, respectively.

Similarly, the ratios of individual pg.belief change increased by 48.79% (HIGH to HIGH), 40.10% (HIGH to LOW), 54.43% (LOW to HIGH), and 35.55% (LOW to LOW) in the anodal group, and the matching ratios were attenuated by 35.00, 38.40, 28.80, and 32.91 % in the cathodal group, respectively (**Figure 6**). The difference in improvement percentage of norm.belief among three stimulation groups with the same identities (HIGH for HIGH and LOW for LOW) is significant [*F*_(2,163)_ = 50.56, *P* < 0.001; Kruskal–Wallis test *P* < 0.001]. The difference among groups with different identities (HIGH for LOW and LOW for HIGH) is also significant [*F*_(2,163)_ = 28.39, *P* < 0.001; Kruskal–Wallis test *P* < 0.001]. The result is basically the same as that for norm.belief.

## Discussion

Resulting *a priori* normative beliefs in a social environment are controlled by a widespread neural network, including the rLPFC, which plays an important role in decision-making. This study investigated the influence of the neurophysiological modulation of rLPFC reactivity by means of tDCS on *a priori* normative beliefs. For this purpose, we administered anodal, cathodal, and sham stimulations on the rLPFC while subjects reported their beliefs of peers. Consistent with our hypothesis, enhancing/suppressing the activity in the rLPFC increased/decreased the level of *a priori* normative beliefs, which were tested by the self-reported contribution in the PG in contrast to the sham stimulation. Our results demonstrate that alterations of rLPFC activity can change *a priori* normative beliefs and consequently provide a causal link between rLPFC activity and *a priori* normative beliefs in voluntary cooperation.

Consistent with the results of previous research (Spitzer et al., 2007; Ruff et al., 2013; Liu et al., 2017), we also verified that rLPFC is involved in the neural mechanisms that support social cooperation. This finding is not a coincidence, as the rLPFC is a crucial brain region that is involved in the process of social norms, not only under the enforcement of sanctions based on the UG, but also under voluntary cooperation based on the PG. The former is fair norm and the latter is cooperation norm, and both belong to social norms. In addition, the present experiment sought to test the possible role of rLPFC in beliefs about voluntary cooperation norm followed by others. Ruff et al. (2013) measured some beliefs (i.e., the perceived fairness of the offer and the punishment expected) that the participants held. In their experiment, subject (Player A) was observed while he made decisions about how much of a monetary endowment to split with another participant (Player B). On the baseline condition, Player B could not punish Player A if he deemed the amount of the split to be unfair. On the punishment condition, Player B was permitted to punish Player A if he deemed the offer unfair. However, they did not measure the beliefs separately or directly assess the participants’ beliefs for each treatment condition (Sanfey et al., 2014). Fortunately, our experimenters measured the *a priori* normative beliefs separately for two identities (HIGH player and LOW player) and for all colleagues in each treatment. This design enabled us to directly assess the participants’ beliefs about social norms. Simultaneously, unlike our research based on the PG frame, Ruff et al. (2013) was based on the UG. UG is a kind of zero-sum game where the decision-making status of the proposer and the responder are unequal, which is not conducive to cooperation. Taken together, these differences may be the main factors that contributed to the varying results of the different research frameworks.

There is a growing interest in cognitive science and neuroscience in studying the effect of *a priori* beliefs on behavioral performance and their underlying neural mechanisms (Friston, 2010; Clark, 2013; Hohwy, 2013; Allen and Friston, 2018). What do the brain’s *a priori* beliefs arise from? As Bowles (2004) suggested, there were two potential sources: one source was genes (inherited from our parents) and the other was cultural inheritance (our past experience through learning or gain). For example, a belief general prevails within certain embodied and environmental conditions in the generative sense (Allen and Friston, 2018). Heuristically, if participants were endowed with the *a priori* beliefs which could help their survival, then they will act in ways that were consistent with that *a priori* beliefs. Specifically, during minimizing prediction error which is imperative for survival, participants may necessarily incorporate self-referential information in the form of *a priori* beliefs and long-term memory to characterize their behaviors (Allen and Friston, 2018). In this process, neuromodulation of post-synaptic gain via neurotransmitters (e.g., dopamine and norepinephrine) are proved to communicate the precision of *a priori* beliefs (Feldman and Friston, 2010; Moran et al., 2013; Kanai et al., 2015).

In our experiment, the “right” contribution is self-reported rather than exogenous, that is, it is not an exact amount or proportion of the initial endowment. For example, Player A1 may think Player B1 should contribute 10G$, so he would report his belief about Player B1 on the basis of his own judgment. In the research of Ruff et al. (2013), “participants are using a fairness norm of ‘equity,’ whereby the optimal decision would be to split the pot of money equally between both players” (Sanfey et al., 2014, p.173). In general, the belief tested in our study based on PG was derived from the participants’ own judgment about norms, whereas the belief tested in previous research based on UG was derived from external norms. Therefore, the PG without external punishment is more effective than the UG with a punishment constraint in terms of reflecting people’s true beliefs in voluntary cooperation. Punishment can easily trigger negative emotions, which are associated with cognitive control. Neuroscientific findings prove that negative emotions can lead to proactive aggression (Dambacher et al., 2015) and aggressive response (Riva et al., 2015), which may interfere with the original belief. Social cooperation preferences are forced out and beliefs are changed. However, the true intentions underlying PG exert no such negative effects. To a certain extent, this outcome also shows that our research framework based on PG is more suitable than UG for cooperation norm compliance and its attached beliefs. Thus, our research provides a new paradigm for future studies on belief of social norm compliance.

In this paper, an individual think the “right” contribution is the “norm” which is based on widely shared beliefs how individual group members ought to behave in PG game. The “actual” contribution is the “compliance” that an individual truly performed in a PGs game. Participants considered the criteria for the “right” contribution believed by other subjects (norm.belief) based on the judgment that people should behave in the PGs framework. However, it is well-documented that participants might feel others not follow a norm that even if it exists (e.g., subjects contribute less than what they consider as “fair,” Reuben and Riedl, 2013) and will not perform what they considered as “right” in practice. In this situation, participants believe that there is a discrepancy between “right *per se*” and “actually paid by others.”

We used tDCS (Nitsche et al., 2008) in the present study to examine whether the social norm of belief and voluntary cooperation depends causally on neural processing in the previously identified rLPFC region (Spitzer et al., 2007). A methodological contribution of our study is the design that allows direct focus on the subjects’ belief in voluntary cooperation. This design allows for measuring the *a priori* normative beliefs that is applicable in a specific situation and is informative of the voluntary behavior that is related to cooperation norms. For example, it could have been informative to ask participants what they believe the “right” contribution is for HIGH players A1 and A2 in each of the situations. Further analysis of the available data reveals that the same identities are more likely to behave according to the same type rather than to the different types. This phenomenon is called the identity effect, which also confirms the common saying that birds of a feather flock together. Our study is also relevant to the existing experimental economics literature (Kocher et al., 2008; Reuben and Riedl, 2009; Spiller et al., 2016), which usually identifies departures from pure self-interest payoffs by controlling other motivations. Furthermore, the valuable literature does not typically consider norm.beliefs and pg.beliefs in voluntary cooperation through tDCS stimulation. Our results offer support for this distinction with some proof. Both types of *a priori* normative beliefs can be changed by varying the neural excitability of rLPFC with tDCS and are affected in opposite manners.

However, our results only confirm the stimulation effect that tDCS anodal and cathodal stimulations of rLPFC lead to an increase and decrease in the contribution of *a priori* normative beliefs, respectively. We cannot answer why this stimulation leads to the change. Two models are actually possible: (1) tDCS anodal and cathodal stimulations of rLPFC stimulations lead to a change in the actual normative standards or (2) tDCS anodal and cathodal stimulations of rLPFC stimulations lead to no change in the normative value but rather impacts the downstream of the decision-making process, since decisions can also be influenced by other factors (e.g., cognitive ability). Both effects can also happen, and this may be a possible causal mechanism for future research. In addition, other beliefs may also matter in social decision-making (Sanfey et al., 2014). According to some scholars (Adolphs, 2009), three broad categories of beliefs exist: one’s beliefs about the nonsocial environment, one’s beliefs about the social environment and about what others in the group believe or do, and one’s beliefs about one’s self. For instance, people may have second-order beliefs, which reflect what people think their partner expects them to do with the purpose of establishing a reliable image and achieving a well-deserved social identity (Chang et al., 2011). To further examine the specificity of the present effects, other beliefs (such as second- or higher-order beliefs), may be included in future investigations into the effects of norm beliefs.

## Conclusion

Our finding reveals that rLPFC stimulation affects beliefs in the cooperation norm. Anodal tDCS on the rLPFC can improve the contribution of *a priori* normative belief, whereas cathodal tDCS on the rLPFC can deteriorate it. This research is a promising step toward understanding how neurobiological mechanisms are connected to beliefs in cooperation norms.

## Author Contributions

JL and XL designed the experiment. XL, XN, and CZ performed the experiment. XL and XY analyzed the data. XL and SL drew the figures. XL wrote the manuscript. JL, XL, and XY revised the manuscript. All authors approved the final version of the manuscript to be published.

## Conflict of Interest Statement

The authors declare that the research was conducted in the absence of any commercial or financial relationships that could be construed as a potential conflict of interest.

## Footnotes

1 The normative beliefs in this paper are derived from people’s perception of social norm and should be treated as *a priori* beliefs without updating or *a posteriori* beliefs. This paper treat normative beliefs as *a priori* factors and do not investigate how they evolve in the dynamic situation with feedback, as feedback (e.g., average contributions by others and corresponding payoff) on the one hand can decrease illusory ideas, on the other hand it may cause some uncontrollable noise variables (e.g., anchoring effect, Furnham and Hua, 2011).
