# Corrigendum: Transcranial Direct Current Stimulation of the Right Lateral Prefrontal Cortex Changes *a priori* Normative Beliefs in Voluntary Cooperation

**DOI:** 10.3389/fnins.2019.00849

**Published:** 2019-08-16

**Authors:** Jianbiao Li, Xiaoli Liu, Xile Yin, Shuaiqi Li, Pengcheng Wang, Xiaofei Niu, Chengkang Zhu

**Affiliations:** ^1^China Academy of Corporate Governance, Reinhard Selten Laboratory, Business School, Nankai University, Tianjin, China; ^2^Department of Economics and Management, Nankai University Binhai College, Tianjin, China; ^3^School of Business Administration, Zhejiang Gongshang University, Hangzhou, China; ^4^Business School, Tianjin University of Finance and Economics, Tianjin, China

**Keywords:** *a priori* normative beliefs, voluntary cooperation, identity, rLPFC, transcranial direct current stimulation

In the original article, there was an error. The participants in the experiment were insufficiently described.

A correction has been made to the ***Materials and methods***, subsection ***Subjects***:

“The subjects of this experiment were the same as Liu et al. (2017) and Li et al. (2018). A total of 83 healthy subjects (recruited from Nankai University students; 41 females and 42 males ranging from 20 to 30 years old) were kept in the sample. None of them had suffered from any neurological or psychiatric disorders. One participant in the anodal stimulation treatment felt discomfort, and we terminated the experiment. Participants randomly divided into three treatments, namely, cathodal (*n* = 28, 12 males), anodal (*n* = 27, 18 males), and sham (*n* = 28, 12 males) stimulation. All the participants had no ex-ante knowledge of neurological (tDCS) or PG tasks, and all voluntarily joined this study with informed consents. The experiment was performed in accordance with the Declaration of Helsinki and was approved by the Ethics Committee of Business of Nankai University. All these 83 participants reported no adverse side effects (e.g., pain on the scalp or headaches) after the experiment.”

In addition, the experiment procedure was insufficiently described.

A correction has been made to the ***Materials and methods***, subsection ***Tasks and*** ***Procedure***, *paragraph three*:

“The payoff function of PG was $\pi_{\text{i}}={\text{X}}_{\text{i}}-{\text{x}}_{\text{i}}+0.6\overset{4}{\underset{\text{i}=1}{\sum }}{\text{x}}_{\text{i}}$, where X_i_ was the endowment, x_i_ was the contribution, and $\overset{4}{\underset{\text{i}=1}{\sum }}{\text{x}}_{\text{i}}$ was the sum contributions of participants from the same group. At the beginning of each trial, the subjects were informed of their identity types (A1, A2, B1, and B2). Then they were asked to answer questions related to beliefs about themselves, voluntary cooperative level and beliefs about others. We did not focus on the beliefs about themselves and voluntary cooperative level in the current study. However, we have emphatically discussed them in Liu et al. (2017) and Li et al. (2018), respectively. In this paper, we focused on the beliefs about others which were tested by pg.belief questions and norm.belief questions:”

The authors apologize for these errors and state that they do not change the scientific conclusions of the article in any way. The original article has been updated.
